# Diffuse-Type Tenosynovial Giant Cell Tumor: What Are the Important Findings on the Initial and Follow-Up MRI?

**DOI:** 10.3390/cancers16020402

**Published:** 2024-01-17

**Authors:** Woo Suk Choi, Seul Ki Lee, Jee-Young Kim, Yuri Kim

**Affiliations:** Department of Radiology, St. Vincent’s Hospital, College of Medicine, The Catholic University of Korea, Seoul 06591, Republic of Korea

**Keywords:** tenosynovial giant cell tumor, diffuse type, magnetic resonance imaging, local recurrence, osteoarthritis, targeted therapy, treatment response

## Abstract

**Simple Summary:**

Tenosynovial giant cell tumor is a benign yet aggressive neoplasm of the synovium that predominantly affects young patients. The tumor comprises two subtypes: the localized type and diffuse type, with the diffuse type exhibiting significantly higher aggressiveness. MRI stands out as the most valuable imaging modality for both its diagnosis and planning its treatment. When interpreting the initial MRI for suspected tenosynovial giant cell tumor, it is imperative to consider: (i) the characteristic findings of tenosynovial giant cell tumor, (ii) the potential findings of the diffuse type, and (iii) the tumor’s resectability. In interpreting follow-up MRIs of the diffuse type after treatment, it is crucial to consider both local recurrence and the development of early osteoarthritis after surgery as well as the treatment response after systemic treatment. Recognizing the distinctive MRI findings of diffuse type tenosynovial giant cell tumor before and after treatment enhances radiologic evaluation, contributing to optimal patient management.

**Abstract:**

Tenosynovial giant cell tumor (TSGCT) is a rare soft tissue tumor that involves the synovial lining of joints, bursae, and tendon sheaths, primarily affecting young patients (usually in the fourth decade of life). The tumor comprises two subtypes: the localized type (L-TSGCT) and the diffuse type (D-TSGCT). Although these subtypes share histological and genetic similarities, they present a different prognosis. D-TSGCT tends to exhibit local aggressiveness and a higher recurrence rate compared to L-TSGCT. Magnetic resonance imaging (MRI) is the preferred diagnostic tool for both the initial diagnosis and for treatment planning. When interpreting the initial MRI of a suspected TSGCT, it is essential to consider: (i) the characteristic findings of TSGCT—evident as low to intermediate signal intensity on both T1- and T2-weighted images, with a blooming artifact on gradient-echo sequences due to hemosiderin deposition; (ii) the possibility of D-TSGCT—extensive involvement of the synovial membrane with infiltrative margin; and (iii) the resectability and extent—if resectable, synovectomy is performed; if not, a novel systemic therapy involving colony-stimulating factor 1 receptor inhibitors is administered. In the interpretation of follow-up MRIs of D-TSGCTs after treatment, it is crucial to consider both tumor recurrence and potential complications such as osteoarthritis after surgery as well as the treatment response after systemic treatment. Given its prevalence in young adult patents and significant impact on patients’ quality of life, clinical trials exploring new agents targeting D-TSGCT are currently underway. Consequently, understanding the characteristic MRI findings of D-TSGCT before and after treatment is imperative.

## 1. Introduction

Tenosynovial giant cell tumor (TSGCT) is a benign yet locally aggressive neoplastic disorder affecting the synovium, which includes joints, bursae, and tendon sheaths [1]. The disorder encompasses intra-articular and extra-articular manifestations [2]. In the 2013 World Health Organization (WHO) classification of soft tissue and bone tumors, the nomenclature was updated based on the tumor’s growth pattern (Table 1) [3]. The diffuse form of this disease is now referred to as diffuse type TSGCT (D-TSGCT), replacing the term “pigmented villonodular synovitis”, while the more common localized form is characterized as localized type (L-TSGCT), replacing the term “giant cell tumor of the tendon sheath”. D-TSGCT is marked by widespread involvement of the synovium, whereas L-TSGCT often presents as a discrete and encapsulated mass [4]. Histologically, there is no clear histological distinction between the two subtypes; therefore, diagnosis relies on radiological assessment and clinical presentation [5].

TSGCT is a rare neoplasm, with incidence rates of 45 and 5 per million person-years for L-TSGCT and D-TSGCT, respectively (Table 2) [6,7]. It exhibits a female predilection (F:M; 2:1) and primarily affects a relatively young patient group, typically aged between 30 and 50 years, although it can occur at any age [6,7]. L-TSGCT constitutes the most common form and is located extra-articularly in 90% of cases. It frequently involves the tendon sheaths of the volar aspect of fingers (85%), followed by locations in the foot and knee (15%) [1,8]. The knee is the most common site when presenting as intra-articular mass [8]. This type typically manifests as a painless, slowly growing soft tissue mass without joint dysfunction [1]. On the other hand, D-TSGCT originates predominantly in the intra-articular space as a unilateral, mono-articular process, most commonly affecting large joints such as the knee (70%), followed by the hip (15%), ankle, shoulder, and elbow [6,9]. In joints affected by D-TSGCT, the synovium becomes hyperproliferative and hemorrhagic, leading to distortion and damage to adjacent osseous, cartilaginous, and tendinous structures [6]. This results in episodes of hemarthrosis, pain, swelling, and, in later stages, severe osteochondral destruction and localized tissue damage [7]. Notably, most cases of extra-articular form of D-TSGCT are believed to represent extensions of primary intra-articular disease [10].

Histologically, the disease is characterized by inclusions of multinucleated giant cells, macrophages, and hemosiderin [11]. L-TSGCT typically presents a multinodular, well-delineated process embedded in a dense, partially collagenous pseudocapsule. In contrast, D-TSGCT lacks a collagenous pseudocapsule and exhibits a diffuse infiltrative sheet-like growth pattern along the synovium, featuring cleft-like spaces and discohesive zones [3]. The two subtypes of TSGCT share a common underlying pathogenesis, primarily associated with a colony-stimulating factor 1 (CSF1) translocation resulting in CSF1 overexpression. This overexpression leads to an increase in neoplastic cells by binding to CSF1 receptors (CSF1R) and accumulating CSF1R-presenting cells [12]. Achieving complete resection can be challenging, particularly in cases of extensive tumor growth. Incomplete resections are associated with a higher likelihood of tumor recurrence [13,14]. In the case of intra-articular D-TSGCT, treatment involves wide local excision, total synovectomy, or arthroplasty, but it carries a high local recurrence rate, reaching up to 50%, often with multiple recurrences [8]. Extra-articular D-TSGCT also exhibits recurrence in 33% to 50% of cases, frequently with multiple recurrences (Table 2) [10]. In the absence of sarcomatous transformation, it is exceedingly rare for D-TSGCTs to develop distant metastasis, with only a few cases thus far [15,16]. Sarcomatous changes, whether de novo or metachronous at presentation, are an exceedingly rare phenomenon in both intra-articular and extra-articular forms of D-TSGCTs [17].

Given the prevalence of D-TSGCT in young adult patents and its significant impact on their quality of life, it becomes crucial to become acquainted with the distinctive magnetic resonance imaging (MRI) findings associated with D-TSGCT both before and after treatment. This review article aims to comprehensively examine the MRI features of D-TSGCT before and after treatment, with the overarching goal of enhancing radiologic evaluation for the optimal management of patients.

## 2. MRI Findings for D-TSGCT on Initial MRI

### 2.1. MRI Protocols for TSGCT

In the diagnosis and treatment of TSGCT, MRI stands out as the most distinctive imaging technique [18]. The scanning protocol for soft tissue tumors should encompass, at a minimum, the following sequences: one set of T1-weighted images (T1WI) before the administration of gadolinium chelate, one set of T2-weighted images (T2WI) with or without fat-suppression techniques (such as fat-saturation, fluid sensitive, DIXON method, or short tau inversion recovery sequences), and one set of T1WI following the injection of gadolinium chelate, with fat-suppression [19]. Intravenous gadolinium contrast facilitates tumor detection and proves useful for follow-up after synovectomy [1]. A gradient echo (GRE) sequence may also be beneficial for detecting hemosiderin related to tumor bleeding [1].

### 2.2. Signal Intensity (SI) for D-TSGCT

**Figure 1:**
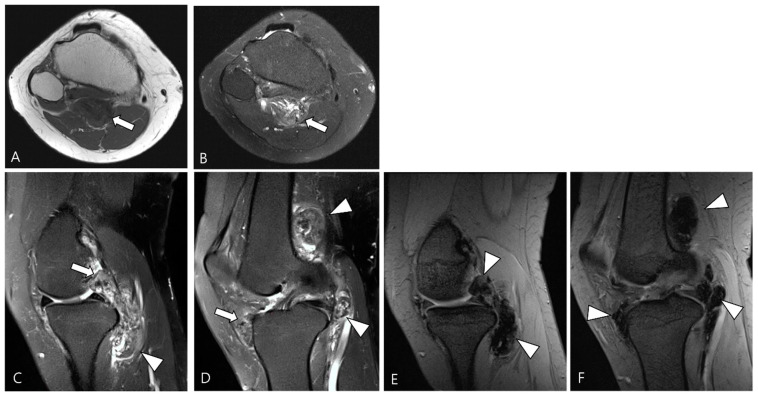
A 46-year-old female with D-TSGCT in the knee. (**A**) Axial T1WI shows iso-to-low SI mass (arrow). (**B**) Axial fat-suppressed T2WI shows heterogeneously high SI (arrow). (**C**,**D**) Sagittal fat-suppressed T2WI images show nodular thickening of synovium containing low SI foci (arrows) with extra-articular extension (arrowheads). (**E**,**F**) Sagittal GRE sequences show blooming artifact due to hemosiderin deposition along the synovium (arrowheads).

The SI of TSGCT on MRI exhibits heterogeneity. T1WI reveals a hypointense to isointense SI, while T2WI displays a hyperintense SI with areas of low SI (Figure 1) [1]. D-TSGCT is prone to bleeding, with bleeding being more common than in L-TSGCT. Numerous prior studies have indicated that D-TSGCT exhibits more robust hemosiderin deposition compared to L-TSGCT, presumably attributed to more frequent occurrences of bleeding and inflammation in D-TSGCT, relative to L-TSGCT [10,20,21,22]. Consequently, extensive hemosiderin deposition is a frequent observation in D-TSGCT, manifesting as low SI within the tumor on both T1WI and T2WI (Figure 1) [1].

Hemorrhage serves as a classic imaging hallmark of D-TSGCT, typically identified as a blooming artifact on GRE sequences (Figure 1). The blooming artifact represents a paramagnetic susceptibility artifact resulting from hemosiderin deposition, characterized by the enlargement and disproportionately lower SI of blood deposits on GRE images compared to spin echo sequences [1]. However, the presence of the blooming artifact is not exclusive to TSGCT [21]. Conditions such as synovial hemangioma and hemophiliac arthropathy may present similar findings on GRE image [3].

**Figure 2:**
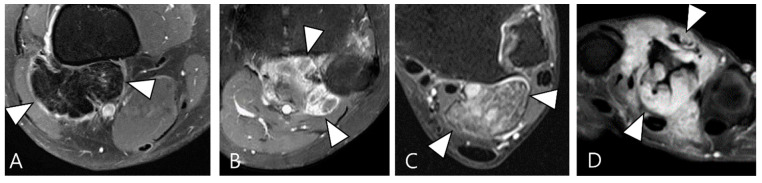
Enhancement patterns of pathology-proven D-TSCGT. (**A**) No contrast enhancement within the lobulated mass (arrowheads) in the posterior knee. (**B**) Peripheral contrast enhancement of the mass with a sparing central portion (arrowheads) in the posterior knee. (**C**) Heterogenous contrast enhancement of the mass (arrowheads) in the posterior ankle. (**D**) Homogenous contrast enhancement of the mass (arrowheads) surrounding the third metacarpal bone.

TSGCT demonstrates enhancement following gadolinium administration [23]. The presence of numerous proliferative capillaries in the collagenous stroma contributes to the strong enhancement in TSGCT [23,24]. Moderate contrast enhancement in TSGCT was reported in 48% of cases by Huang et al. [25]. However, the contrast enhancement is nonspecific, spanning a broad spectrum, including lack of enhancement, peripheral enhancement, heterogeneous enhancement, and homogeneous enhancement (Figure 2) [21,26,27]. A previous study noted variable degrees of contrast enhancement between D-TSGCT and L-TSGCT without statistical significance [28].

### 2.3. Morphological Findings for D-TSGCT

**Figure 3:**
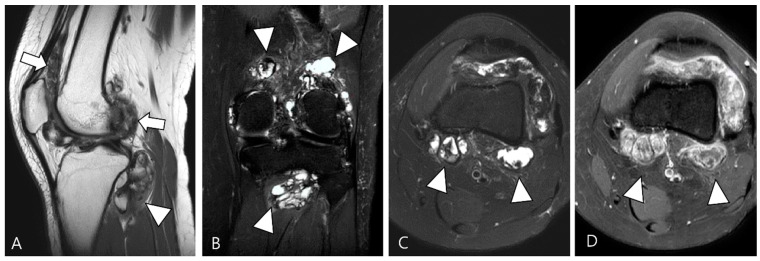
A 27-year-old female with D-TSGCT in the knee. (**A**) Sagittal T2WI shows diffuse and multinodular synovial thickening with scattered dark SI foci (arrows) in the knee joint space. Extra-articular extension to the popliteus myotendinous junction is noted (arrowhead). (**B**,**C**) Coronal and axial fat-suppressed T2WIs show that proliferated synovium engulfs the reactive joint effusion resulting in multichambered cystic mass-like lesions around popliteal fossa (arrowheads). (**D**) Axial contrast-enhanced T1WI reveals thickened synovium as diffuse septal enhancement within the cystic changes (arrowheads).

TSGCT can exhibit variable morphological appearances on MRI, depending on its anatomical location and growth patterns [3]. Intra-articular forms of D-TSGCT are prone to diffuse spreading, adopting a multicompartmental growth pattern that involves at least two contiguous intra-articular synovial recesses. The characteristic findings include irregular synovial thickening (>5 mm), often described as “frond-like” with villous or nodular morphology. This synovial proliferation tends to envelop associated reactive joint effusion, leading to the formation of multiloculated thick-walled trapped cystic masses (Figure 3) [1]. Kim et al. [28] conducted a study on various MRI morphologic parameters for D-TSGCT, including nodularity and margin. Nodularity was classified according to Al Qattan as type I, representing “a single round or multilobulated tumor” or type II, indicating “two or more distinct, separated tumors” [29]. The margin was categorized as “circumscribed” when the lesion’s border was clearly delineated from surrounding structures and as “infiltrative” if the lesion’s borders were indistinguishable from the surrounding structures [30]. The study revealed that intra-articular D-TSGCT manifests as multinodular masses scattered throughout the joint, with multinodularity potentially serving as the sole indicator for determining mass features in intra-articular D-TSGCT (Figure 3) [28].

**Figure 4:**
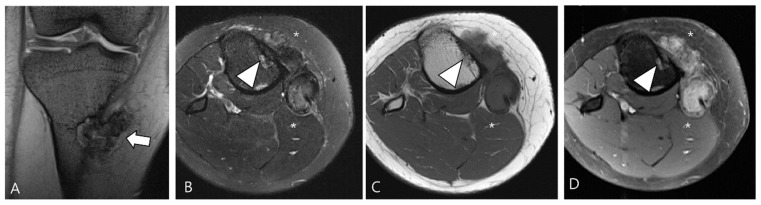
A 40-year-old female with D-TSGCT in the tibia. (**A**) Coronal GRE sequence shows a lobulated heterogenous SI mass in the juxtacortical area of the proximal metaphysis of the tibia with blooming artifact (arrow). Note that there is no remarkable intra-articular communication of the knee joint. (**B**) Axial fat-suppressed T2WI, (**C**) T1WI, and (**D**) fat-suppressed enhanced T1WI show the mass arising from the Pes anserine bursa with focal bony erosion of the adjacent tibia (arrowheads) and infiltration to the adjacent muscle and subcutaneous fat layer (asterisks).

**Figure 5:**
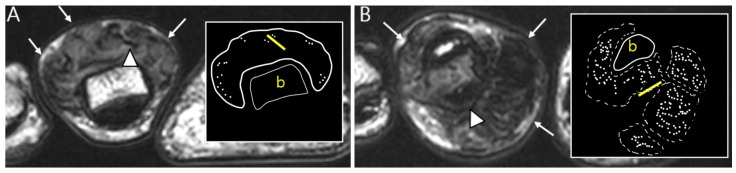
Nodularity, margin, and peripheral hypointensity between two subtypes. (**A**) Pathology-proven L-TSGCT of the foot shows that the mass is shown as a single mass with a circumscribed margin (arrows) on axial T2WI. The mass shows the avid peripheral hypointensity (see in box) and encasement of the extensor tendon (arrowhead). (**B**) Pathology-proven D-TSGCT of the foot shows that the masses contain multiple distinct nodules with an infiltrative margin from the surrounding tissues (arrows) on axial T2WI. The masses show the absent peripheral hypointensity (see box) and encasement of the flexor tendon (arrowhead). Box; b = bone, yellow line = tendon.

The extra-articular growth of D-TSGCT primarily arises as a result of extra-articular extension of intra-articular D-TSGCT through transcapsular fenestrations (Figure 1 and Figure 3) [31]. However, due to its infiltrative growth pattern, determining the origin is often challenging [32]. Instances of extra-articular D-TSGCT without intra-articular communication are exceedingly rare, and such cases involve the bursa around the knee, such as popliteal bursa (Figure 4) [20,32,33,34,35]. In cases where TSGCTs occur in the digits, they are typically located in extra-articular portion, and it is crucial to differentiate between two subtypes [36]. Jeong et al. [36] conducted a comparison of the MRI morphologic features of TSGCTs in the digits between the diffuse type and localized type. Their findings revealed that D-TSGCTs in digits manifest as multinodular and infiltrative masses without a peripheral capsule. Kim et al. [28] also found that the most sensitive MRI parameter with the highest odds ratio for extra-articular D-TSGCT, which was the lack of peripheral hypointensity (lack of capsule) (Figure 5).

### 2.4. Relationship to Adjacent Structures of D-TSGCT

D-TSGCT typically exhibits infiltration into adjacent structures [32]. MRI serves as a valuable tool for detecting the relationship to anatomical or surgical landmarks, with key parameters including (i) articular or cartilage involvement, (ii) bone involvement, (iii) muscular/tendinous/ligamentous tissue involvement, and (iv) neurovascular involvement [18].

**Figure 6:**
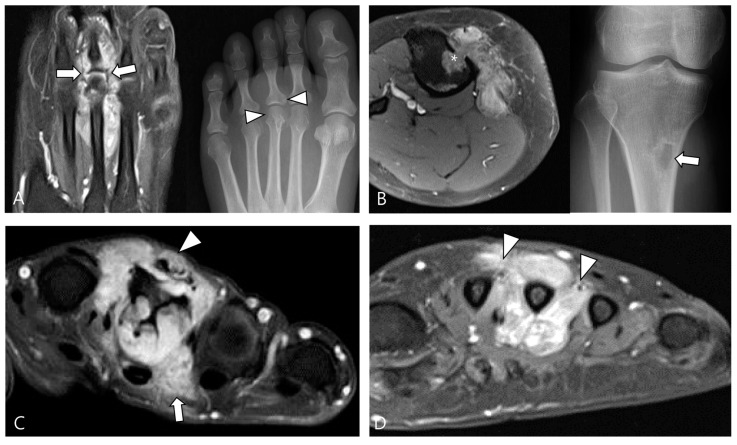
Involvement of adjacent structures of D-TSGCT on contrast-enhanced fat-suppressed T1WIs. (**A**) Articular involvement (arrows) of the metatarsophalangeal joint of the foot, also correlated on a plain radiograph (arrowheads) presenting as a periarticular bony change. (**B**) Bone erosion (asterisk) of the tibia, also correlated on a plain radiograph (arrow). (**C**) Tendon (arrowhead) and muscular involvement (arrow) with infiltration into the extensor tendon fibers and interosseous muscle of the foot. (**D**) Neurovascular bundle involvement (arrowheads) with more than 180° encasement of the neurovascular bundle of the foot.

Articular or cartilage involvement (Figure 6A) is defined as the mass infiltrating the synovial lining of a small joint or causing cartilage defect/thinning by extending into a large joint [28]. Bone involvement (Figure 6B) often manifests as extrinsic erosion with well-defined sclerotic margins. These extrinsic erosions can be notably deep, simulating bone marrow invasion with an aggressive process, although this feature is rare [3]. The prevalence of bone erosion depends substantially on the site of involvement, specifically the joint capacity [3]. Larger capacity joints like the knee, allowing extension and decompression of normal tissue into multiple adjacent bursal regions, are less frequently affected by extrinsic erosion of bone. In contrast, smaller capacity joints such as the hip, shoulder, elbow, and ankle are more likely to demonstrate extrinsic erosion of bone [3]. Muscular/tendinous/ligamentous tissue involvement (Figure 6C) is defined as the mass infiltrating into fibers [36]. Neurovascular involvement (Figure 6D) is characterized by mass involvement of more than 180° of a neurovascular bundle [36].

Articular or cartilage involvement demonstrates the highest specificity for diagnosing D-TSGCT, irrespective of intra-articular or extra-articular location [28]. Jeong et al. [36] conducted a comparison of the disease extent of TSGCTs in the digits between the diffuse type and localized type, revealing that D-TSGCTs exhibit more severe disease extents of articular/cartilage and muscle/tendon involvement than L-TSGCT [36]. The comparison of MRI findings between L-TSGCT and D-TSGCT is summarized in Table 3.

In the case of the knee, a clinical classification using MRI has been introduced for both diagnosis and treatment decisions (resectability) [37]. In this study [37], the relationship with adjacent structures was assessed, resulting in three types and four subtypes. This was presented as a simple and easy-to-use clinical classification. Type 1 (localized type), controlling the tumor and restoring knee function can be achieved through simple resection. Arthroscopic tumor resection is advised for subtype 1a (localized type in the joint capsule), while direct tumor resection is recommended for subtype 1b (localized type outside the joint capsule). Since bone involvement by D-TSGCT is not uncommon, intra-articular D-TSGCT (type 2) of the knee included two subtypes. Complete tumor resection with a single incision or combined anterior and posterior incisions is recommended for patients with intra-articular D-TSGCT without bone involvement (subtype 2a), while for intra-articular D-TSGCT with bone involvement (subtype 2b), complete tumor resection of both soft tissue and bone is advised. For D-TSGCT spanning both the inside and outside of the joint capsule (type 3), achieving complete tumor resection was nearly impossible and neo-adjuvant or adjuvant therapies are recommended due to the high risk of recurrence after surgery alone.

### 2.5. Advanced MRI Sequences for D-TSGCT

Advanced MRI techniques, such as diffusion weighted imaging (DWI) and dynamic contrast-enhanced imaging (DCE), have the potential to significantly improve the imaging-based diagnoses for musculoskeletal tumors [38]. The increased cellularity observed in malignant tumors leads to restricted diffusion, which is reflected in a lower apparent diffusion coefficient (ADC) value [39]. Tumor-related angiogenesis and hypervascularity contribute to rapid arterial enhancement, a phenomenon detectable through DCE [38].

**Figure 7:**
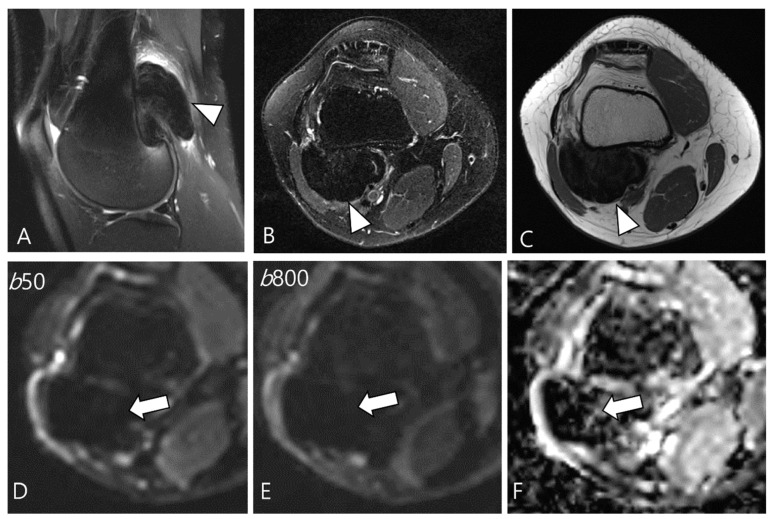
A 29-year-old female with D-TSGCT in the distal femur. (**A**,**B**) Sagittal and axial fat-suppressed T2WIs show a low SI mass at the popliteal fossa (arrowheads). (**C**) Axial T1WI also shows low SI due to extensive hemosiderin deposition (arrowhead). (**D**,**E**) The mass shows low SI on low and high b-value images of DWI due to hemosiderin deposition (arrows). (**F**) The mass creates a pseudo-low ADC value on the ADC map (arrow), suggesting T2 black-out effect.

**Figure 8:**
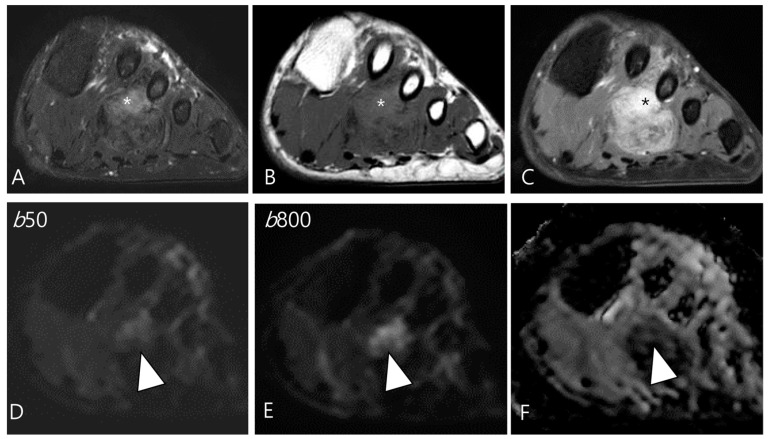
A 46-year-old male with D-TSGCT in the foot. (**A**) Axial fat-suppressed T2WI shows a heterogeneously high (asterisk) to low SI soft tissue mass involving the second web space. The mass extends to the plantar muscles. (**B**) Axial T1WI shows the mass with iso (asterisk) to low SI. (**C**) Axial contrast-enhanced fat-suppressed T1WI shows the heterogenous enhancement (asterisk) within the mass. (**D**,**E**) The enhancing portion of the mass shows persistent high SI on both the low and high b-value images of the DWI (arrowhead). (**F**) The mass shows a low ADC value (arrowhead) in this area, suggesting diffusion restriction.

Ashikyan et al. [27] conducted a study on DWI encompassing giant cell tumor (GCT) of bone and TSGCT, which are two histologically distinct neoplasms with overlapping characteristics. Despite their different histopathological appearances, a common cell lineage is proposed, supported by a study that explored the ultrastructural cytochemical features of cells in bone, tendon sheath, and intra-articular GCTs, revealing similarities of tartrate-resistant acid phosphatase (TRAP)-positive cells in all three tumor types [40]. The study further observed that osseous GCTs and TSGCTs exhibit similar and low ADC values [27,39]. Commonly, there is a belief that malignant tumors tend to exhibit lower ADC values, while benign tumors typically display higher ADC values [41,42]. However, TSGCT may present the T2 black-out effect, resulting in a pseudo-low ADC value of less than 1.0 × 10^−3^ mm^2^/s due to the presence of hemosiderin and other blood products (Figure 7) [39,43]. Other potential reasons for the low ADC values of GCTs include the nature of the intralesional matrix or the presence of hypercellular components [27]. (TS)GCT comprises giant osteoclast-like cells interspersed with a hypercellular and vascularized stroma, and this composition can influence the lower ADC measurement (Figure 8) [44].

**Figure 9:**
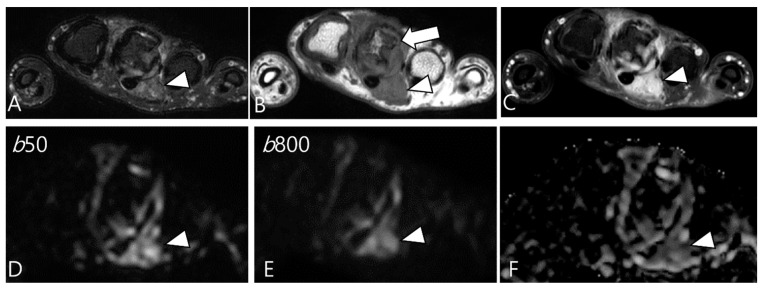
A 37-year-old female with D-TSGCT in the hand. (**A**) Axial fat-suppressed T2WI shows lobulated soft tissue with heterogenous SI around the third MCP joint (arrowhead). (**B**) Axial T1WI shows the mass with iso SI (arrowhead) and marked extrinsic bony erosion at the metacarpal bone (arrow) is noted. (**C**) Axial contrast-enhanced fat-suppressed T1WI shows the homogeneous enhancement of the mass (arrowhead). (**D**,**E**) Due to microcapillary perfusion, the mass shows an apparent high SI on the low b-value image of the DWI (arrowhead) compared to the high b-value image of the DWI. (**F**) The mass shows a low ADC value (arrowhead), suggesting diffusion restriction.

Moreover, their fractional ADC analysis revealed several intriguing findings: ADC calculations from low b-value pairs were higher than the ADC calculations from high b-value pairs, a phenomenon thought to be associated with hyperperfusion in these neoplasms [27,45]. In fact, DWI obtained with b-values below 200–400 s/mm^2^ is influenced by tissue microcapillary perfusion [46]. Ashikyan et al. [27] reported that GCTs become apparent at low b value images, known to be hyperperfused (Figure 9). This characteristic can be illustrated by DCE as a time-intensity curve showing rapid early enhancement with a plateau phase [47].

## 3. Differential Diagnoses for D-TSGCT on Initial MRI

Most synovial diseases predominantly exhibit heterogeneously high SI on T2WI. Nevertheless, within daily clinical practice, we encounter a subset of synovial lesions manifesting low SI on T2WI. Such lesions often exhibit distinct characteristics and predilections, allowing for a more tailored approach to the differential diagnosis [48]. The presence of T2 hypointensity on synovial diseases can be attributed to various factors, and their specific characteristics may align with conditions such as TSGCT, along with synovial chondromatosis, rheumatoid arthritis, chronic tophaceous gout, amyloid arthropathy, and hemosiderotic synovitis (Table 4) [48].

### 3.1. Differential Diagnoses of Intra-Articular D-TSGCT

On MRI, intra-articular D-TSGCT manifests as a mass-like synovial proliferation with infiltrative margins and may extend extensively throughout the synovial lining [49]. The lesions tend to bleed, leading to hemosiderin deposition, which is evident as a decrease in SI across all pulse sequences [49]. The primary considerations for the differential diagnosis involve lesions that also demonstrate low SI on T2WI, attributable to various factors such as blood components of different stages, calcification, inorganic crystals, fibrosis, and/or amyloid deposition [24,48,49,50].

#### 3.1.1. Hemosiderotic Synovitis

**Figure 10:**
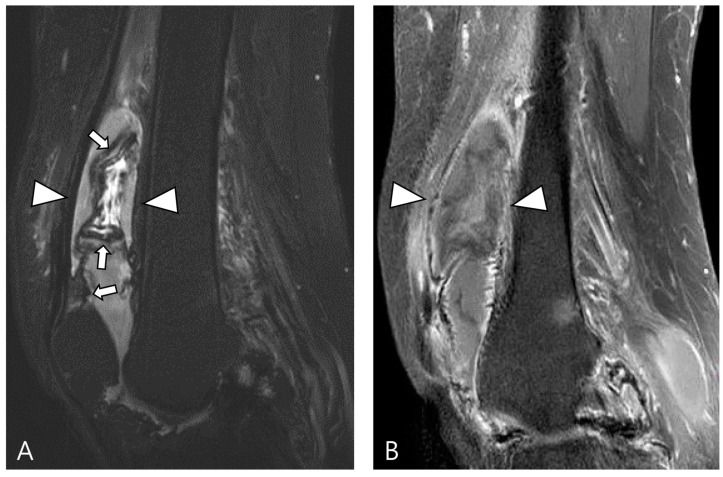
A 67-year-old female with hemosiderotic synovitis in the knee. (**A**) Sagittal fat-suppressed T2WI shows a large amount of joint effusion in the suprapatellar recess (arrowheads). The synovium is diffusely thickened with a dark SI lining (hemosiderin deposition, arrows). (**B**) Sagittal contrast-enhanced fat-suppressed T1WI shows poor contrast enhancement on the distended suprapatellar bursa (arrowheads).

Hemosiderotic synovitis is a rare proliferative synovial disorder resulting from chronic recurrent intra-articular bleeding [51]. The primary cause of such bleeding is hemophilia, while other contributing factors include osteoarthritis, chronic trauma, rheumatoid arthritis, anticoagulant use, hemochromatosis, and myeloproliferative disease [51]. In the chronic stage of the disease, it is characterized by lateral dominant osteoarthritis and/or lateral meniscus injury in the elderly [49]. Hemosiderotic synovitis and intra-articular D-TSGCT may present similar clinical and radiological features, with both conditions exhibiting a blooming artifact on GRE images [52]. Despite the resemblance in MRI findings, hemosiderotic synovitis tends to involve knee and demonstrates a lesser degree of contrast enhancement compared to the D-TSGCT, particularly in the distribution within the suprapatellar bursa where the maximum thickness of the synovium is observed (Figure 10) [51].

#### 3.1.2. Synovial Chondromatosis

**Figure 11:**
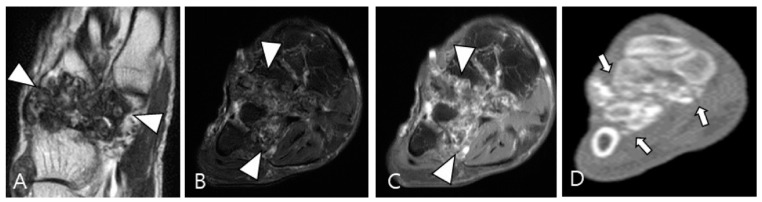
A 54-year-old male with synovial chondromatosis in the foot. (**A**) Coronal T2WI demonstrates diffusely low SI of synovial thickening at the Lisfranc or Chopart joints (arrowheads). (**B**) Axial fat-suppressed T2WI reveals infiltrative soft-tissue extension into the surrounding bone and muscles (arrowheads). (**C**) Axial contrast-enhanced fat-suppressed T1WI shows minimal peripheral enhancement (arrowheads). (**D**) Axial CT reveals multiple small conglomerated calcifications in involved tarsometatarsal joints (arrows) with bony erosion.

Synovial chondromatosis arises from self-limiting proliferative and metaplastic changes in the synovium [49,53]. It is categorized into primary and secondary forms; the primary form is currently considered a benign neoplastic disease based on cytogenetic analyses, while the secondary form is associated with underlying joint abnormalities such as osteoarthritis, trauma, or previous infectious or inflammatory arthritis [48]. Monoarticular involvement is common, with the knee being the most frequently affected joint, followed by the hip, elbow, shoulder, and ankle [48]. The condition can present as multiple round bodies, similar in size and shape, with MRI revealing these loose bodies amidst synovial proliferation [49,54]. Mineralized loose bodies exhibit low SI on all pulse sequences, while non-mineralized areas show low SI on T1WI and high SI on T2WI, reflecting the increased water content of hyaline cartilage [48]. Plain radiography or computed tomography (CT) is the imaging modality of choice for identifying cartilaginous nodules, which display a cobblestone pattern and varying degrees of calcification (85% is calcified) or ring-and-arc patterns of mineralization (Figure 11) [48,53]. In long-standing diseases, peripheral enchondral ossification or central dystrophic mineralization can develop within the loose bodies [48,53]. The presence of calcification or metaplastic cartilage helps differentiate synovial chondromatosis from D-TSGCT.

#### 3.1.3. Dialysis-Related Amyloid Arthropathy

**Figure 12:**
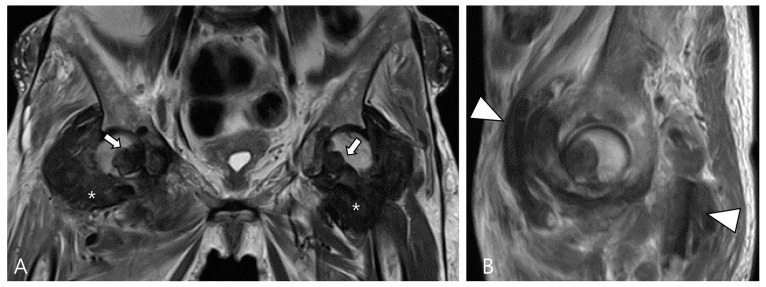
A 62-year-old female with amyloid arthropathy in both hip joints. (**A**) Coronal T2WI shows periarticular soft tissue masses (asterisks) extending to intra-articular space with heterogeneously low SI in both hip joints. Bony erosions at both femoral heads (arrows) are combined. (**B**) Sagittal T2WI shows that the mass infiltrates into the adjacent tendon and muscles (arrowheads).

Amyloid arthropathy is a complication associated with long-term hemodialysis [49]. It arises from the deposition of a distinctive form of amyloid, derived from circulating β2-microglobulin, within the synovial fluid, bone, and periarticular tissues. Increased serum levels of β2-microglobulin in hemodialysis patients result from the ineffectiveness of both hemodialysis and peritoneal dialysis membranes in filtering this substance [49,50]. Commonly affected joints include the shoulder, hip, femur, and knee [55]. On MRI, amyloid deposition exhibits a heterogeneously low SI on both T1WI and T2WI, which can resemble intra-articular D-TSGCT [50]. Despite the short T2 relaxation time characteristic of amyloid-containing tissue due to their hypocellular and fibrous nature, amyloid deposition does not demonstrate a paramagnetic effect on GRE sequences [49]. While D-TSGCT typically manifests as monoarticular arthropathy, amyloid arthropathy presents as symmetric polyarthritis with joint or periarticular swelling, accompanied by tendinous amyloid deposition in periarticular areas (Figure 12) [49]. Notably, a clinical history spanning more than 5 years since the initiation of hemodialysis can be a crucial diagnostic clue [55].

#### 3.1.4. Chronic Rheumatoid Arthritis

**Figure 13:**
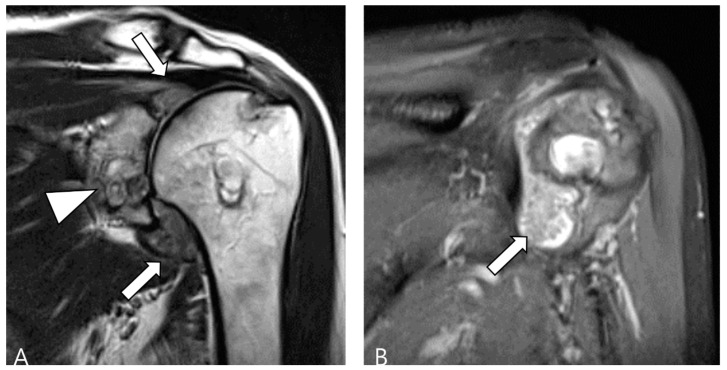
A 60-year-old female with rheumatoid arthritis of the shoulder. (**A**) Coronal T2WI demonstrates cystic and erosive change in the glenoid rim (arrowhead) with extensive synovial proliferation in the glenohumeral joint (arrows). (**B**) Coronal fat-suppressed T2WI shows multiple tiny low SI foci, known as ‘rice bodies’, within the hyperplastic synovium (arrow).

Rheumatoid arthritis, a chronic autoimmune inflammatory disorder primarily affecting synovial tissues and joints, is characterized by a proliferative, hyperplastic, hypervascular, and locally invasive synovial reaction termed pannus [54]. The condition typically presents as bilateral symmetrical polyarthritis, with a predilection for the small joints [48]. On MRI, synovitis is characterized by increased water content and includes numerous inflammatory cells, granulation tissue, and abundant blood vessels, resulting in high SI on T2WI with robust contrast enhancement [48]. In the late stage, synovial fibrosis gradually evolves, manifesting as a turbid fluid showing hypointensity with poor contrast enhancement [56]. Additionally, rice bodies, which are multiple loose bodies of approximately the same size and shape, represent a characteristic finding in rheumatoid arthritis (Figure 13) [56].

#### 3.1.5. Tophaceous Gout

**Figure 14:**
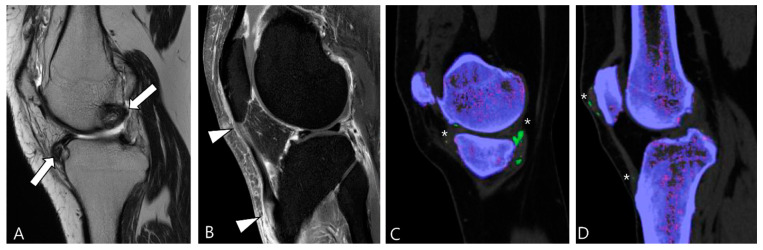
A 39-year-old male with tophaceous gout of the knee. (**A**) Sagittal T2WI shows heterogeneously low SI masses along the synovial lining of the knee joint (arrows). (**B**) Sagittal contrast-enhanced fat-suppressed T1WI shows the abnormal enhancement with nodular thickening of patellar tendon (arrowheads). (**C**,**D**) Dual-energy CT reveals MSU crystal deposition (asterisks) with green color coding along the synovial linings and quadriceps-patellar aponeurosis. Trabecular or cancellous bone is displayed in purple.

Gout, an inflammatory response triggered by the deposition of monosodium urate (MSU) crystals as a result of hyperuricemia, evolves into its chronic phase known as tophaceous gout, characterized by asymmetric polyarthritic distribution [48]. While tophi are commonly periarticular, they can also involve the articular or bursal synovium [49]. In MRI, intra-articular tophi typically exhibit heterogeneously low SI on both T2WI and T1WI, a feature that may resemble intra-articular D-TSGCT [57]. The degree of enhancement varies due to hypervascular granulation tissue surrounding the tophus or inflammatory tissue within the tophi [54]. Distinguishing gout from D-TSGCT can be aided by typical locations such as the quadriceps, patellar tendon, and Achilles tendon (Figure 14) [49]. Although tophi may show mineralization, calcification is not usually apparent in radiographs [48]. Recently, dual-energy CT has emerged as an alternative noninvasive diagnostic tool for identifying and quantifying MSU crystals (Figure 14) [58,59].

### 3.2. Differential Diagnoses of Extra-Articular D-TSGCT

Extra-articular D-TSGCT is thought to signify the extension of primary intra-articular disease through transcapsular extension [31]. Purely extra-articular D-TSGCT is rare in the literature and likely originates most frequently from the synovium of the bursae and tendon sheath [20,35,36]. Extra-articular D-TSGCT manifests a local destructive growth pattern, extensively infiltrating and entrapping adjacent soft tissue while often eroding bone [8]. It can manifest as an infiltrative soft tissue mass with low SI on both T1WI and T2WI due to hemosiderin deposition [50].

#### 3.2.1. Fibroma of the Tendon Sheath (FTS)

**Figure 15:**
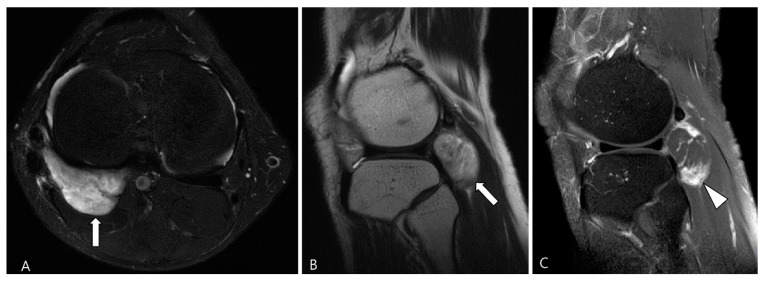
A 44-year-old male with FTS of the knee. (**A**,**B**) Axial fat-suppressed T2WI and sagittal T2WI show a well-defined high-SI mass containing strip-like low SI bundles at the popliteal fossa (arrows). (**C**) Sagittal contrast-enhanced fat-suppressed T1WI shows an ovoid-shaped mass with septal enhancement (arrowhead).

FTS is a rare benign tumor commonly found in the hands, wrists, and feet, similar to TSGCT [56]. Despite the similarities in MRI features between FTS and TSGCT, Ge et al. [22] identified key differences. The patterns of low SI in the two tumor types vary: FTS exhibits strip-like or disordered low SI at the center of the lesion, likely due to the presence of dense collagen fiber bundles with all hypointense areas, possibly because most spindle cells are concentrated in the center (Figure 15). In contrast, TSGCT predominantly displays granular or separated low SI, located near the periphery of the tumor, as foam cells that can engulf hemosiderin, resulting in patchy or nested distribution around the lesion. The morphology of the two types also differs: FTS tumors are predominantly round or ovoid (Figure 15), while TSGCT tumors are cast-molding and lobulated [22,56,60]. Although both FTS and TSGCT have been reported to cause absorption of adjacent bone, only a few cases of FTS with bone erosion have been described [61]. TSGCT is more likely to cause extensive bone erosion and surrounding destruction [62].

#### 3.2.2. Extra-Abdominal Desmoid-Type Fibromatosis (DF)

**Figure 16:**
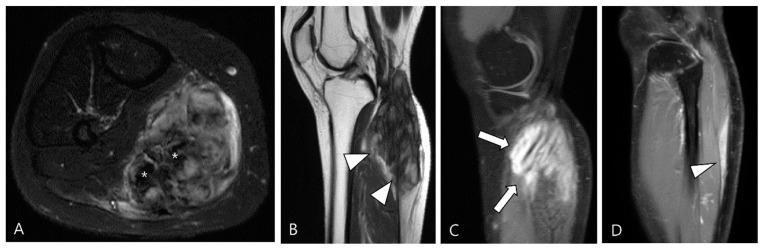
A 40-year-old female with extra-abdominal DF in the lower leg. (**A**) Axial fat-suppressed T2WI shows a slightly hyperintense intramuscular mass with intralesional dark SI portions (asterisks) in the medial head of the gastrocnemius muscle. (**B**) Sagittal T2WI shows this lesion with a lobulated contour with an infiltrative margin (arrowheads). (**C**,**D**) Sagittal contrast-enhanced fat-suppressed T1WIs show that this mass displays heterogenous enhancement and finger-like tumor extension into adjacent muscle (termed as “staghorn sign”, arrows) and a tapering appearance of the tumor extension along the fascia (termed as “fascial tail sign”, arrowhead).

DF is a locally aggressive (myo)fibroblastic neoplasm that originates in deep soft tissues, characterized by infiltrative growth and a propensity for local recurrence without metastatic potential [63,64]. Aggressive fibromatosis, musculoaponeurotic fibromatosis, and desmoid tumor are synonymous term for DF [63]. The major subgroups include superficial (palmar and plantar) and deep fibromatoses. The deep fibromatoses are further classified as extra-abdominal (found in the upper extremities, lower extremities, trunk, head and neck), abdominal wall (arising from musculoaponeurotic structures of the abdominal wall), and intra-abdominal (in the mesentery or pelvis) [63,65]. Extra-abdominal DF originates from the connective tissue of muscle and their overlying aponeurosis or fascia, potentially infiltrating adjacent subcutaneous tissue and muscle [65]. DF exhibits a heterogeneous appearance on MRI, with variable SI on T2WI and T1WI due to diverse intralesional components, including myxoid matrix, cellular stroma, and fibrous tissue/collagen bands [66,67]. Decreased T2 SI corresponds to dense collagen and hypocellularity, while increased T2 SI correlates with high cellularity [66]. TSGCT and extra-abdominal DF exhibit similar SI on MRI, but DF manifests along the fascial planes, with or without muscle invasion. Recognizing the characteristic signs of extra-abdominal DF aids in distinguishing between the two diseases (Figure 16); DF displays the staghorn sign (fingerlike tumor extension into muscle or subcutaneous fat) and fascial tail sign [68].

#### 3.2.3. Tophaceous Gout

**Figure 17:**
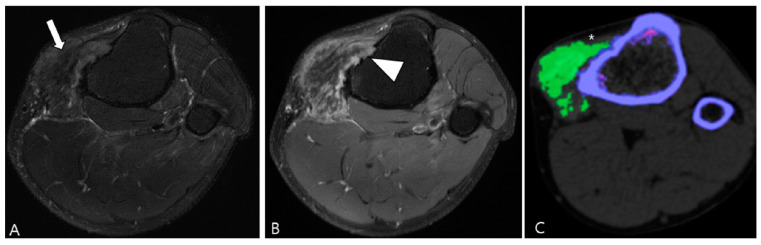
A 47-year-old male with tophaceous gout of the knee. (**A**) Axial fat-suppressed T2WI reveals a lobulated extra-articular slightly hyperintense soft tissue mass containing low SI foci along the Pes anserine bursa (arrow). (**B**) Axial contrast-enhanced fat-suppressed T1WI shows heterogeneous enhancement and focal cortical erosion (arrowhead) at the proximal medial tibia. (**C**) Dual-energy CT reveals the lesion is tophi with MSU deposition of green color coding (asterisk). Trabecular or cancellous bone is displayed in purple.

Gouty tophi predominantly manifest as periarticular nodules [49]. Tophi are soft tissue masses that appear hypointense on T2WI, potentially resembling D-TSGCT [50]. However, gouty tophi can depict cortical erosions, marrow edema and variable signal characteristics depending on the amount of calcium present on the MRI [58]. Radiographs can be useful in evaluating soft tissue calcifications, and dual-energy CT may be employed to confirm the presence of MSU crystals in the tophi (Figure 17) [69].

## 4. MRI Findings for D-TSGCT on Follow-Up MRI

### 4.1. Treatment Options for D-TSGCT

The objective in treating intra-articular D-TSGCT is to remove all abnormal synovium, thereby preventing local recurrence and ultimately reducing the risk of osteoarthritis [70]. Treatment of extra-articular D-TSGCT is essential to prevent the destruction of the affected tendon or bursa [71]. Available treatment options encompass surgical resection, radiation therapy, pharmaceutical modulation of the disease, or a combination of these approaches [3].

Surgical excision remains the primary treatment for TSGCT [3]. However, the long-term success of surgery hinges on the ability to achieve complete disease resection [3]. Logically, a cure is more achievable in the L-TSGCT, which exhibits low recurrence rates (Table 2). Conversely, for D-TSGCT, the surgical approach is contentious, marked by a high recurrence rate (Table 2) [1,37,72]. The surgical strategy for intra-articular D-TSGCT depends on the involved joint, disease extent, and surgeon’s experience and preference [3]. Striking a balance between complete tumor resection and preservation of joint function is challenging [37]. Synovectomy may be conducted through either an arthroscopic or open arthrotomy technique; regardless of the approach, complete disease resection is imperative to minimize the recurrence rates [3]. While arthroscopic surgery offers the advantage of minimal functional loss and quicker rehabilitation, these benefits must be weighed against the potential incompleteness of diseased tissue resection [73]. Open arthrotomy with synovectomy enhances the likelihood of complete disease resection but typically entails immobilization and a more prolonged recovery with significant postoperative stiffness [71,74].

Radiation therapy can be employed as the primary treatment for intra-articular D-TSGCT, but its optimal use is as a complement to surgery in cases of incomplete disease resection [3]. Radiation can be delivered through external beam or via intra-articular injection of radioactive isotopes, a technique known as radiosynoviorthesis [75]. Minimal side effects, such as erythema, have been reported, and patients generally tolerate the therapy well without skin breakdown [3]. Theoretical concerns include the potential development of malignancy, either in the synovium or bone, following external radiation therapy [3].

Expression of colony-stimulating factor 1 (CSF1) gene expression was found to be elevated in cases of TSGCT, and CSF1 is implicated in the proliferation and differentiation of neoplastic cells, activating cells of the mononuclear, phagocytic lineage [76]. These monocytic cells constitute the giant cells characteristic of TSGCT [12,76]. Structure-guided blockade of the CSF1-receptor (CSF1R) kinase has been employed, leading to prolonged regression in tumor volume in most patients [77]. Pexidartinib, offering a novel non-surgical treatment option, was developed to address intra-articular D-TSGCT [37,78]. Results from the ENLIVEN trial, a randomized, placebo-controlled phase III trial, demonstrated that Pexidartinib therapy not only led to a decrease in tumor volume but also resulted in an improvement in the range of motion, leading to FDA approval for treatment [9,79].

### 4.2. Checklists on Follow-Up MRI for D-TSGCT

MRI plays a crucial role in follow-up for D-TSGCT [1]. Table 5 summarizes the checklists on follow-up MRI according to the types of treatment.

**Figure 18:**
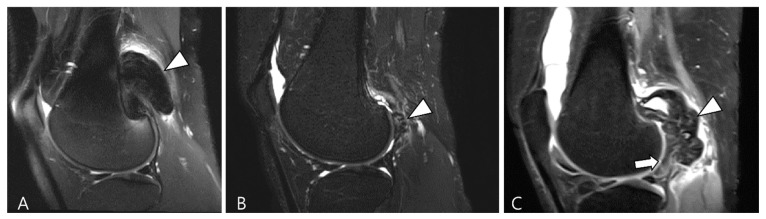
Serial T2WI follow-ups for D-TSGCT. (**A**) Initial MRI shows a low SI mass at the popliteal fossa (arrowhead). (**B**) After En bloc excision, the first follow-up MRI (1 year) shows small low SI nodule at the posterior joint capsule (arrowhead). (**C**) Second follow-up MRI (3 years) shows recurrent mass extra-articularly (arrowhead) and extensive nodular thickening intra-articularly (arrow).

In terms of surgical outcome, local recurrence is defined as the presence of new disease after synovectomy or the observation of growing residual disease on a follow-up MRI scan [18]. Diffuse synovial thickening within the first 6 months can be equivocal for residual disease due to associated reactive synovitis. However, suspicion of disease recurrence should arise if there is evidence of growing, enhancing solid, and nodular synovial thickening (Figure 18) [1].

**Figure 19:**
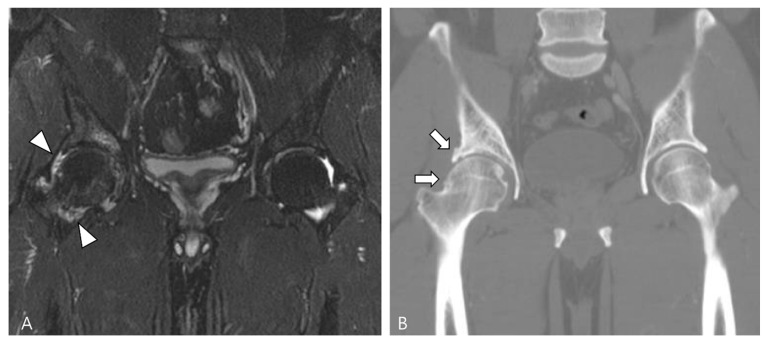
A 47-year-old male with D-TSGCT in the right hip joint. (**A**) Initial coronal fat-suppressed T2WI shows a tiny low SI nodular synovial thickening in the right hip joint (arrowheads). (**B**) After synovectomy, follow-up CT after 4 years shows the development of osteoarthritis (arrows) in the right hip joint.

D-TSGCT also poses a risk for the early development of osteoarthritis, particularly in the knee, hip, and ankle [80]. Recurrent disease is likely to necessitate multiple surgeries, leading to significant joint morbidity that could expedite the degenerative process associated with D-TSGCT toward secondary osteoarthritis (Figure 19) [81]. Lin et al. [81] reported that nearly 30% of patients underwent at least two surgeries during follow-up, and the risk of recurrent surgery was twice as high in patients with osteoarthritis compared with those without. This underscores the imperative for effective non-surgical treatment options for D-TSGCT, especially in patients with secondary osteoarthritis [81].

Radiotherapy has been recommended as either an adjuvant to surgery or as the primary treatment for inoperable patients [82]. Nevertheless, several scholars have investigated the efficacy and side effects of traditional radiotherapy techniques [83]. Considering that D-TSGCT patients are typically young, there is a notable concern regarding the long-term risks, encompassing malignant transformation, fibrosis, joint stiffness, and other potential sequelae [83]. While there have been reported cases treated with the recent advanced technique such as image-guided intensity-modulated radiotherapy [82], published reports on this matter are generally limited, especially on MRI findings. Prospective studies are necessary to gain a better understanding of the potential role of this treatment modality for D-TSGCT patients. Though there are no published data, if a patient with D-TSGCT who has undergone radiotherapy undergoes an MRI scan, it would be necessary to assess not only local recurrence but also consider skin necrosis and the possibility of malignant transformation [82].

While D-TSGCT exhibits neoplastic features with clonal cytogenetic abnormalities, it shares many characteristics with inflammation related to rheumatoid arthritis. D-TSGCT is likely situated in an intermediate state between an inflammatory and a neoplastic process [84]. Therefore, despite surgery being the primary treatment option, emerging systemic treatments targeting CSF1R are gaining prominence, and MRI is essential for objectively assessing treatment response [85,86]. The quantification of tumor volume change serves as a crucial parameter for evaluating treatment response [1]. Peterfy et al. [87] introduced a semiquantitative, joint-specific visual tumor volume score (TVS) for D-TSGCT, expressing tumor volume as a percentage of the estimated volume of the maximally distended normal synovial cavity of the involved joint. However, since TVS is a semiquantitative tool, its reproducibility has limitation [1]. Beyond changes in tumor size, pilot studies have described other specific MRI findings following CSF1R inhibitors. These findings include a decrease in SI with a reduction in capsular distension and joint effusion, as well as an increase in hemosiderin deposition [2]. These findings may be particularly valuable for patients on Pexidartinib, where the overall tumor burden remains essentially stable on serial MRIs, and changes in SI and hemosiderin deposition may be the only imaging findings indicative of a positive treatment response [2]. These imaging features seem to correlate well with clinical improvements, such as pain reduction [79]. Hemosiderin scars, referred to as a low SI lining along the synovium after therapy, may persist without corresponding clinical complaints [87]. It has been suggested to use term “complete response” in case of residual hemosiderin scars with a short axis <5 mm [87].

## 5. Conclusions

A multidisciplinary approach is essential to enhance outcomes for patients dealing with recurrent and refractory disease of D-TSGCT, and this approach should incorporate meticulous MRI evaluation before and after treatment [78]. MRI stands out as the preferred modality for diagnosing D-TSGCT, planning surgeries with adjuvant radiotherapy or systemic targeted therapy, and assessing local recurrence following surgery or treatment response to systemic therapies. We emphasize the imaging characteristics of D-TSGCT alongside potential mimickers and assess checklists following various treatment options.

## Figures and Tables

**Table 1:** WHO classification (2013) of TSGCT according to its growth pattern.

Localized Type	Diffuse Type
A more common form affecting only a portion of the synovium	A less frequent form involving substantial parts of the synovium
Predominantly involving the digits and wrists	Primarily affecting large joints (knee, hip, ankle, elbow)
Systematically benign	More aggressive and destructive and may exceptionally include a malignant component

**Table 2:** Incidence and recurrence rate of TSGCT.

	Localized Type	Diffuse Type
Incidence	45 per million person-years	5 per million person-years
Recurrence rate	<15%	50% with intra-articular disease 33 to 50% with extra-articular disease

**Table 3:** Comparison of MRI findings between L-TSGCT and D-TSGCT.

MRI Findings	Localized Type TSGCT	Diffuse Type TSGCT
Signal intensity on T1WI, T2WI and contrast enhancement	Hypo-to-iso SI on T1WI and heterogeneous SI on T2WI with variable degrees of contrast enhancement	
GRE images	Presence of blooming artifact due to hemosiderin deposition	
Low SI areas (due to bleeding and hemosiderin deposition)	Speckled	Extensive and granular
Nodularity	Single	Multinodular
Margin	Circumscribed	Infiltrative
Peripheral hypointensity (capsule)	Present	Absent
Articular or cartilage involvement	Not frequent	Frequent
Bone involvement	Uncertain difference between two subtypes	
Muscle or tendon involvement	Not frequent	Frequent
Neurovascular involvement	Uncertain difference between two subtypes	

**Table 4:** Differential diagnoses of T2 low SI disease with clinical importance.

Location	Disease	Key Component	MR Imaging Features	Clinical Importance
Intra-articular	Intra-articular D-TSGCT	Hemosiderin	Extensive synovial thickening with bleeding Blooming artifact on GRE images	Systematically benign
	Hemosiderotic Synovitis	Hemosiderin	Blooming artifact on GRE images Tends to involve knee Lesser contrast enhancement	Chronic recurrent intra-articular bleeding Hemophilia, osteoarthritis, chronic trauma, rheumatoid arthritis, etc.
	Synovial Chondromatosis	Calcification	Mineralized loose bodies show low SI on all pulse sequences Non-mineralized areas show low SI on T1WI and high SI on T2WI	Primary form: benign neoplastic disease Secondary form: associated with underlying joint abnormalities such as osteoarthritis, trauma, etc. Plain radiography for cartilaginous nodules
	Dialysis-Related Amyloid Arthropathy	Amyloid	Heterogeneously low SI on T1WI/T2WI No paramagnetic effect on GRE images	History of more than 5 years of hemodialysis
	Chronic Rheumatoid Arthritis	Rice bodies	Synovial hyperplasia Turbid fluid with T2W low rice bodies	Chronic autoimmune inflammation with pannus formation
	Tophaceous Gout	Tophi	Heterogeneously low SI on T1WI/T2WI	Hyperuricemia Dual-energy CT for MSU crystals
Extra-articular	Extra-articular D-TSGCT	Hemosiderin	Transcapsular extension of primary intra-articular disease with tumor infiltration Peripheral granular or separated low SI	More aggressive and destructive and may exceptionally include a malignant component
	Fibroma of the Tendon Sheath	Dense collagen	Round or ovoid shape Strip-like or disordered central low SI	Benign tumor with slow growth
	Extra-abdominal Desmoid-Type Fibromatosis	Fibrous tissue/collagen bands	Variable SI on T2WI/T1WI between cellularity and fibrous tissue Staghorn sign Fascial tail sign	Locally aggressive and infiltrative neoplasms
	Tophaceous Gout	Tophi	Cortical bony erosions with marrow edema Variable signal SI depending on the amount of calcium percentage	On chronic stage, presents as asymmetric polyarticular distribution Dual-energy CT for MSU crystals

**Table 5:** Checklists on follow-up MRI by the types of treatment.

Treatment Types	Checklists on Follow-Up MRI
Surgical excision	• Local recurrence
	• Early development of osteoarthritis
Radiotherapy	• Local recurrence
	• Skin necrosis
	• Malignant transformation
CSF1-receptor inhibitors	• Semiquantitative tumor volume change
	• Decrease in SI along synovium with reduction in capsular distension and joint effusion
